# A comparison of special intrauterine balloons and intrauterine contraceptive devices in the treatment of intrauterine adhesions

**DOI:** 10.1007/s00404-023-06993-y

**Published:** 2023-03-10

**Authors:** Lin-Lin Zhuang, Kai Wang, Hai-Lan Shen, Jia-Hui Lin, Ye Lu, Zhen-Yu Luo, Wen-Rong Wang

**Affiliations:** 1 Department of Family Planning, Women and Children’s Hospital, School of Medicine, Xiamen University, Siming District, Zhenhai Road, Xiamen, 361005 Fujian China; 2 Department of Gynecology, Women and Children’s Hospital, School of Medicine, Xiamen University, Xiamen, 361005 Fujian China

**Keywords:** Intrauterine adhesions, Special intrauterine balloon, Intrauterine contraception device, Hysteroscopy

## Abstract

**Purpose:**

This study aimed to compare the efficacy of a special kind of intrauterine balloon (IUB) and that of an intrauterine contraception device (IUD) for patients with intrauterine adhesions (IUAs) after transcervical resection of adhesion (TCRA).

**Methods:**

In this retrospective cohort study, after TCRA, 31 patients received a special IUB, and 38 patients received an IUD. The Fisher exact test, logistic regression method, Kaplan–Meier method and Cox proportional hazards regression model were used for statistical analysis. A two-sided value of *P* < 0.05 was considered statistically significant.

**Results:**

The readhesion rate significantly differed between the IUB group and IUD group, at 15.39% and 54.06%, respectively (*P* = 0.002). For recurrent moderate IUA, patients in the IUB group had lower scores than patients in the IUD group (*P* = 0.035). There was a significant difference in the intrauterine pregnancy rate of IUA patients in the IUB group and IUD group after treatment, with rates of 55.56% and 14.29%, respectively (*P* = 0.015).

**Conclusion:**

Patients in the special IUB group had better outcomes than those in the IUD group, which has a certain guiding significance for clinical work.

## What does this study add to the clinical work?

**Table:** 

IUA patients treated with the special IUB after TCRA have a better outcome than IUD.	

## Introduction

Intrauterine adhesions (IUAs), also known as Asherman syndrome, refer to partial or total adhesion of the uterine cavity or cervical canal due to endometrial trauma. The assessment and diagnosis of IUAs may include hysterosalpingograpy (HSG), hysteroscopy, transvaginal ultrasonography, between them, hysteroscopy is the gold standard [1]. There are a variety of clinical manifestations, including menstrual abnormalities, such as hypomenorrhea, amenorrhea, dysmenorrhea, infertility, recurrent spontaneous abortion (RSA), placenta previa or placental implantation. Studies have shown that there is a rising trend in the prevalence of IUAs in China, and dilation and curettage (D&C) is the most common cause of IUAs, especially uterine curettage after termination in different periods of pregnancy. According to statistics, the incidence of IUAs after multiple D&C procedures is as high as 14.0–32.0% [2, 3].

The treatment of Asherman syndrome aims to restore the anatomy and volume of the uterine cavity, improve the symptoms of different clinical presentations, and, most importantly, prevent the reformation of IUAs and repair the endometrium. Transcervical resection of adhesion (TCRA) by hysteroscopy is a routine operation for IUAs; however, studies have shown that the recurrence rate after TCRA reaches up to 48.0–62.5% and that the pregnancy rate is only 22.5–33.3% [4, 5]. Therefore, it is crucial to take measures to prevent readhesion and promote endometrial regeneration and repair. At present, the methods applied to reduce the IUA recurrence rate after TCRA mainly include the placement of intrauterine balloon (IUBs), intrauterine contraceptive device (IUDs) and biological glue materials. Estrogen, growth factor and granulocyte colony-stimulating factor (G-CSF) can be used to promote endometrial growth. Occasionally, a number of adjuvant drugs, such as aspirin, sildenafil citrate, and pentoxifylline, are used to improve uterine artery blood perfusion [6]. A cohort study suggested that the IUB and IUD are superior to biological glue in preventing readhesion after surgery; in addition, the IUB has better efficacy than the IUD [2].

It is of vital importance to prevent the recurrence of IUAs. To date, which method has a higher efficacy is still a matter of controversy. The purpose of this retrospective cohort study was to analyze the difference in curative effects in IUA patients who were treated with a special kind of IUB or IUD after TCRA, which has a certain guiding significance for clinical work.

## Materials and methods

### Patients and data collection

In this retrospective cohort study, 69 patients with Asherman syndrome were treated at Women and Children’s Hospital, School of Medicine, Xiamen University between May 2020 and August 2021. All of the patients experienced TCRA by hysteroscopic surgery. TCRA were performed with two kinds of surgical techniques: mechanical procedures (non-electrical) or electrical management. 31 patients underwent IUB placement in the family planning department totally treated with cold scissors, and 38 patients underwent IUD placement in the gynecology department treated with cold scissors or electricity. The follow-up cutoff time was October 2021. Clinical data were gathered from the institutional database. All patients met the following inclusion criteria: (1) after TCRA, only placement of IUB or IUD to prevent adhesions recurrence, without multiple intrauterine placement; (2) the operations were all successfully accomplished using the same operating systems (6.5 mm hysteroscopy, Olympus company, Tokyo, Japan); (3) no contraindication to use estrogen-progesterone sequential therapy. All of the clinical records were anonymized. The approval for this study was provided by the ethics committee of Women and Children’s Hospital, School of Medicine, Xiamen University, Xiamen, China.

### Evaluation before TCRA

Prior to TCRA, all patients underwent preoperative evaluations consisting of a detailed medical history, especially a history of menstrual patterns, reproductive history, previous intrauterine operation history, clinical examinations, routine blood and vaginal secretion determination, electrocardiography and transvaginal ultrasonography. In this paper, RSA is defined as ≥ 2 spontaneous abortions with the same sexual partner.

### Operation

**Fig. 1:**
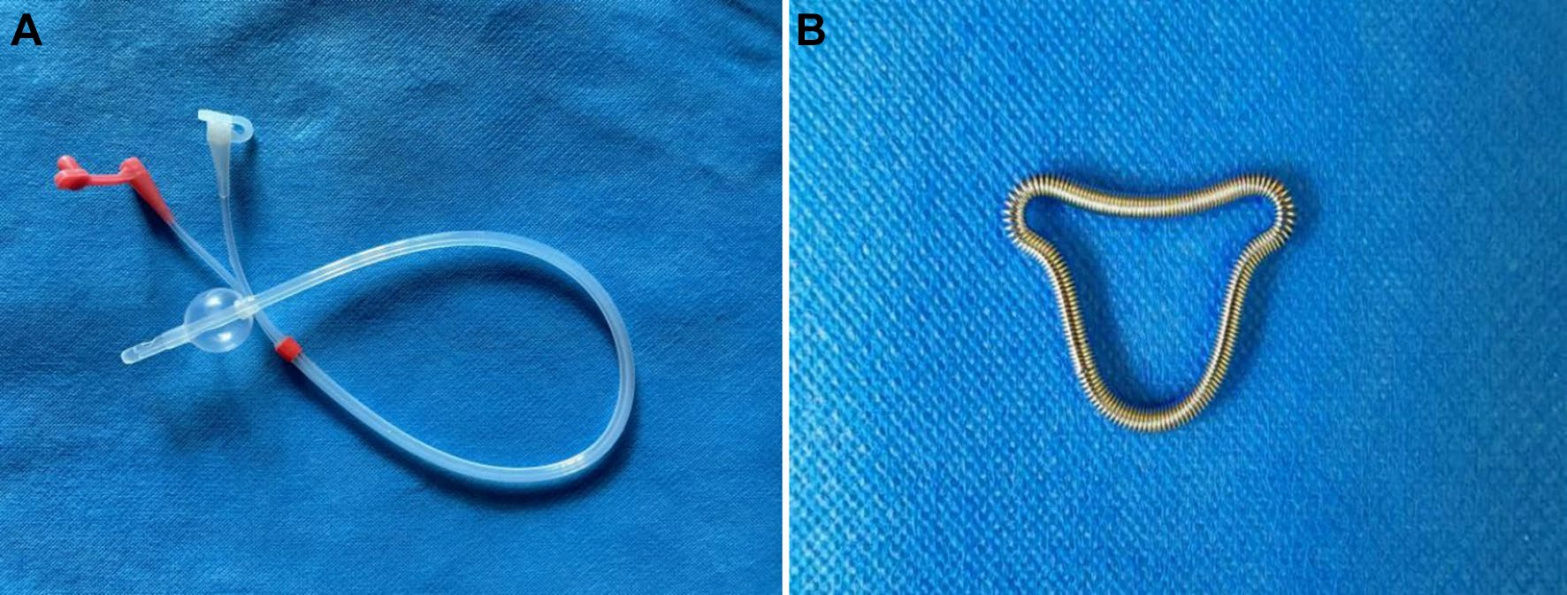
Special intrauterine ballon (**A**), heart-shaped copper IUD (**B**)

In all cases, transcervical resection of adhesion by hysteroscopy was performed under general anesthesia. After adhesion separation, patients involved in this study took either of the two adjunctive treatments (a special IUB or a heart-shaped copper IUD, Fig. 1) as a readhesion preventive measure. The intrauterine balloon used in this study is unique in its much softer elastic saccule (4.0 millimeter/mm, F12, Manufacturer: Nantong Sanli Medical Devices Co., Ltd.) that is inflated with 2.5–3.0 milliliters (ml) normal saline; in addition, it is attractive in price and quality. Usually, a tube with this kind of balloon is applied for hydrotubation or HSG. At the end of the procedure, the extra tube outside the cervix was curled up into the vagina, keeping the orifice open to drain fluid.

Chronic endometritis (CE) is a continuous inflammation of the endometrium, and there are many plasma cells in the endometrial stroma. As a marker, CD138 and CD38 are detected to find plasma cells by immunohistochemistry (IHC) [7–9]. In this study, patients were diagnosed with CE based on their postoperative pathologic results.

According to the 1988 American Fertility Society (AFS) scoring system [10], patients with IUAs are divided into three stages: mild (a score of 1–4), moderate (a score of 5–8) and severe (a score of 9–12). This classification standard evaluates the extent of cavity involvement, texture of adhesion and menstrual pattern of patients during hysteroscopic surgery. In this study, IUAs were classified into three types as (1) central type, an adhesion where there was space between the adhesion and uterine wall; (2) peripheral type, an adhesion located on the uterine wall or fundus; (3) mixed type.

### Treatment after TCRA

All 69 patients with Asherman syndrome had undergone postoperative hormone sequential therapy with estrogen-progesterone based on the last menstrual period. From the day of surgery, complex packing estradiol tablets/estradiol and dydrogesterone tablets (Femostone, 2 mg/mg, 2 mg, 10 mg. Manufacturer: Abbott Health care Products B.V.) was administered orally. Some gynecologists preferred to administer Progynova (estradiol valerate tablets, 2–6 mg daily, Manufacturer: DELPHARM Lille S.A.S.) for 21 days, during the last 10 days, combined with Duphaston (dydrogesterone tablets, 20 mg daily, Manufacturer: Abbott Health care Products B.V.). Short-term oral prophylactic antibiotic therapy was used routinely. The IUB was taken out 7 days after TCRA. The second-look hysteroscopic procedure was recommended one month after the operation and 2–3 days after menstruation. Nevertheless, some patients were delayed, and five patients in the IUB group did not return for evaluation, and in the IUD group, one patient did not undergo the second-look hysteroscopy. There were no treatment-related complications were found.

### Statistical analysis

The statistical analysis was processed by the SPSS 20.0 statistical software tool for Windows (IBM Corporation, Armonk, NY, USA). The statistical description varied from categorical data and quantitative data. The normality test of continuous variables was performed by the Kolmogorov‒Smirnov Z test. Continuous data that fit the normal distribution are described as the mean ± SD (standard deviation); otherwise, they are described as the median (interquartile range/IQR). Categorical data are described as frequencies and percentages. For different comparisons between the two groups, Fisher’s exact test was used in the univariate analysis, and the logistic regression method was applied for further verification. The cumulative conception rate of patients pursuing pregnancy was calculated by the Kaplan–Meier method and compared by the log-rank test. Then, a Cox proportional hazards regression model was used to calculate hazard ratios (HRs) with 95% confidence intervals (CIs). A two-sided value of *P* < 0.05 was considered statistically significant.

## Results

### Baseline clinical features

Tables 1 and 2 show the clinical characteristic features of 69 patients with Asherman syndrome involved in this study in detail. According to Table 1, IUA patients were classified as three degrees: mild, moderate and severe, in 5 patients (7.25%), 48 patients (69.56%) and 16 patients (23.19%), respectively. The results indicated that age, BMI, number of intrauterine pregnancies, number of D&C procedures, number of intrauterine surgeries, complications with thin endometrium, CE, MA and RSA did not affect the severity of patients with Asherman syndrome (Table 1, *P* > 0.05). There was no significant difference between the moderate IUA patients of the two groups; hence, a comparison of treatment efficiency was made between them. There were 31 patients in the IUB group and 38 patients in the IUD group. According to the difference comparison between the two groups, there were no significant differences between the basic characteristics, such as age, BMI, number of intrauterine pregnancies, number of D&C procedures, number of intrauterine surgeries, symptoms of dysmenorrhea, menstruation assessment before surgery, IUAs types in primary TCRA, complicated with a thin endometrium or not (in this study, a thin endometrium was defined as having a thickness less than 7 mm in the late proliferative and secretory phase [6, 11]), complicated with CE or not, and the interval between two TCRA procedures (Table 2, *P* > 0.05). As shown in Table 2, the mean age of the included patients was 32.65 ± 5.48 years, that is, 32.26 ± 5.38 years of age in the IUB group and 32.97 ± 5.62 years of age in the IUD group. The results indicated that 75.36% (52/69) of the patients experienced a decrease in menstruation, 15.94% (11/69) of the patients experienced amenorrhea, and 8.70% (6/69) of IUA patients experienced dysmenorrhea.

**Table 1:** Univariate correlation analysis of IUA severity

Variables	Mild (*n* = 5)		Moderate (*n* = 48)		Severe (*n* = 16)		*P* value
	*n*	%	*n*	%	*n*	%	
Age (years)							0.294
< 35	3	60.00	32	66.67	7	43.75	
≥ 35	2	40.00	16	33.33	9	56.25	
BMI (kg/m^2^)							0.724
< 18.5	1	20.00	4	8.33	2	12.50	
18.5–24.9	4	80.00	37	77.08	11	68.75	
≥ 25	0	0.00	7	14.58	3	18.75	
Gravidity (IUP)							0.196
0	0	0.00	1	2.08	0	0.00	
1	0	0.00	10	20.83	0	0.00	
≥ 2	5	100.00	37	77.08	16	100.00	
Number of D&C procedures							0.192
0	0	0.00	4	8.33	0	0.00	
1	0	0.00	15	31.25	2	12.50	
≥ 2	5	100.00	29	60.42	14	87.50	
Number of intrauterine surgeries^a^							0.565
0	0	0.00	4	8.33	0	0.00	
1	0	0.00	11	22.92	2	12.50	
≥ 2	5	100.00	33	68.75	14	87.50	
Number of MAs							0.512
0	1	20.00	19	39.58	8	50.00	
≥ 1	4	80.00	29	60.42	8	50.00	
Complicated with thin endometrium							0.700
Yes	2	66.67	29	80.56	11	84.62	
No	1	33.33	7	19.44	2	15.38	
Complicated with CE							0.395
Yes	0	0.00	5	17.24	0	0.00	
No	3	100.00	24	82.76	11	100.00	
Complicated with RSA							0.088
Yes	4	80.00	20	41.67	4	25.00	
No	1	20.00	28	58.33	12	75.00	

IUA intrauterine adhesions, BMI body mass index, defined as weight in kilograms divided by the square of the height in meters (kg/m2), IUP intrauterine pregnancy, D&C dilation and curettage, MA missed abortion, CE chronic endometritis, RSA recurrent spontaneous abortion

^a^Number of intrauterine surgeries referred to surgeries before TCRA

**Table 2:** Baseline clinical features of IUA patients

Categories	IUB (*n* = 31)			IUD (*n* = 38)			Mean ± SD/median (IQR)	*P* value
	*n*	%	Mean ± SD/median (IQR)	*n*	%	Mean ± SD/median (IQR)		
Age (years)			32.26 ± 5.38			32.97 ± 5.62	32.65 ± 5.48	0.330
< 35	21	67.74		21	55.26			
≥ 35	10	32.26		17	44.74			
BMI (kg/m^2^)			20.96 (3.89)			21.78 ± 3.05	21.08 (4.26)	0.661
< 18.5	2	6.45		5	13.16			
18.5–24.9	25	80.65		27	71.05			
≥ 25	4	12.90		6	15.79			
Gravidity (IUP)								0.860
0	0	0.00		1	2.63			
1	4	12.90		6	15.79			
≥ 2	27	87.10		31	81.58			
Number of D&C procedures								0.253
0	1	3.23		3	7.89			
1	5	16.13		12	31.58			
≥ 2	25	80.65		23	60.53			
Number of intrauterine surgeries								0.379
0	1	3.23		3	7.89			
1	4	12.90		9	23.68			
≥ 2	26	83.87		26	68.42			
Dysmenorrhea								0.684
Yes	2	6.45		4	10.53			
No	29	93.55		34	89.47			
Menstruation assessment before surgery								0.333
Amenorrhea	7	22.59		4	10.53			
Less	21	67.74		31	81.58			
Equal	3	9.68		2	5.26			
Increased	0	0.00		1	2.63			
Complicated with a thin endometrium^a^								1.000
Yes	17	80.95		25	80.65			
No	4	19.05		6	19.35			
Complicated with CE								0.153
Yes	3	23.08		2	6.67			
No	10	76.92		28	93.33			
Interval between two times of TCRA (days)			31.50 (11.00)			35.00 (36.00)	33.00 (28.00)	0.439
21–28	10	34.46		9	24.32			
29–56	11	42.31		17	45.95			
> 56	5	16.13		11	29.73			
Menstruation assessment after primary TCRA								0.554
Increased	28	90.32		31	81.58			
Less	1	3.23		1	2.63			
Equal	2	6.45		6	15.79			
IUA score of the secondary TCRA								0.002
0	22	84.62		17	45.95			
2–4	3	11.54		4	10.81			
5–8	1	3.85		14	37.84			
9–12	0	0.00		2	5.41			
Score decrease in moderate IUA patients after the primary TCRA^b^								0.035
≤ 4	2	8.70		8	36.36			
> 4	21	91.30		14	63.64			
IUAs types of moderate patients in primary TCRA								0.094
Central type	8	30.77		2	9.10			
Peripheral type	8	30.77		13	59.09			
Mixed type	10	38.46		7	31.81			
Surgical technique in primary TCRA								< 0.001
Mechanical	31	100.00		14	36.84			
Electric	0	0.00		24	63.16			
Fertility intention								
Contraception	13	41.94		15	41.67			1.000
Pursuing pregnancy	18	58.06		21	58.33			
Pregnancy after the last TCRA								0.015
Yes	10	55.56		3	14.29			
No	8	44.44		18	85.71			

*IUA* intrauterine adhesion, *IUB* intrauterine balloon, *IUD* intrauterine device, *SD* standard deviation, *IQR* interquartile range, *BMI* body mass index, defined as weight in kilograms divided by the square of height in meters (kg/m^2^), *IUP* intrauterine pregnancy, *D&C* dilation and curettage, *CE* chronic endometritis, *TCRA* transcervical resection of adhesion

^a^Thin endometrium was discussed as endometrial thickness less than 7 mm in late proliferative and secretory phase in this study

^b^According to the data difference in distribution, we only analyzed score decrease in moderate IUA patients after primary TCRA

As shown in Tables 1 and 2, 84.06% (58/69) of IUA patients had at least 2 intrauterine pregnancies, 69.57% (48/69) of the patients had at least 2 D&C procedures, 75.36% (52/69) of the patients had at least 2 intrauterine surgeries, and 60.87% (42/69) of the patients had a thin endometrium before TCRA. In total, 11.63% (5/43) of IUA cases were complicated by chronic endometritis (CE).

### Clinical efficacy

In this research, the clinical efficacy was presented through a menstruation assessment after the primary TCRA, the IUA score of the secondary TCRA and the score decrease in moderate IUA patients after the primary TCRA. No significant difference was found in the menstrual changes in patients in the two groups who received a special IUB or IUD, and the menstrual volume was increased in 90.32% and 81.58% of patients, respectively (Table 2, *P* > 0.05). There was a striking difference in the IUA score of the secondary TCRA, which revealed that the readhesion rate varied between the two groups by 15.39% and 54.06%, respectively (Table 2, *P* = 0.002). As mentioned above, this study only statistically tested the difference in score decrease in moderate IUA patients after the primary TCRA, as shown in Table 2. The severity of patients in the IUB group was significantly reduced (*P* = 0.035). According to Table 3, the multivariate logistic analysis further verified the differences between the two subgroups in terms of the IUA score of the secondary TCRA and the score decrease after the primary TCRA. The odds ratio (OR) and 95% CI were OR 2.342 (95% CI 1.473–3.724, *P* < 0.001) and OR 1.632 (95% CI 1.122–2.375, *P* = 0.010), respectively.

**Table 3:** Multivariate logistic analysis of the difference between two subgroups

Variable	OR	95% CI	*P* value
IUA score of the secondary TCRA	2.342	1.473–3.724	< 0.001
Score decrease after the primary TCRA	1.632	1.122–2.375	0.010

*IUA* intrauterine adhesions, *TCRA* transcervical resection of adhesion, *OR* odds ratio, *CI* confidence interval

In addition, this study performed a different analysis of mechanical procedures or electric management during TCRA surgery in moderate and severe IUA patients in the IUD group. As shown in Table 4, cold knife management had a greater advantage in reducing the IUA score after the primary TCRA in severe IUA patients (*P* = 0.011).

**Table 4:** Difference analysis of the two types of hysteroscopic management in moderate and severe IUA patients (only the IUD group)

Decline in the IUA score after the primary TCRA	Cold knife	Electric knife	*P* value
Moderate IUA patients			0.380
≤ 4	2	6	
> 4	7	7	
Severe IUA patients			0.011
≤ 8	0	10	
> 8	3	1	

*IUA* intrauterine adhesions, *IUD* intrauterine device, *TCRA* transcervical resection of adhesion

### Intrauterine pregnancy rate posttreatment

**Fig. 2:**
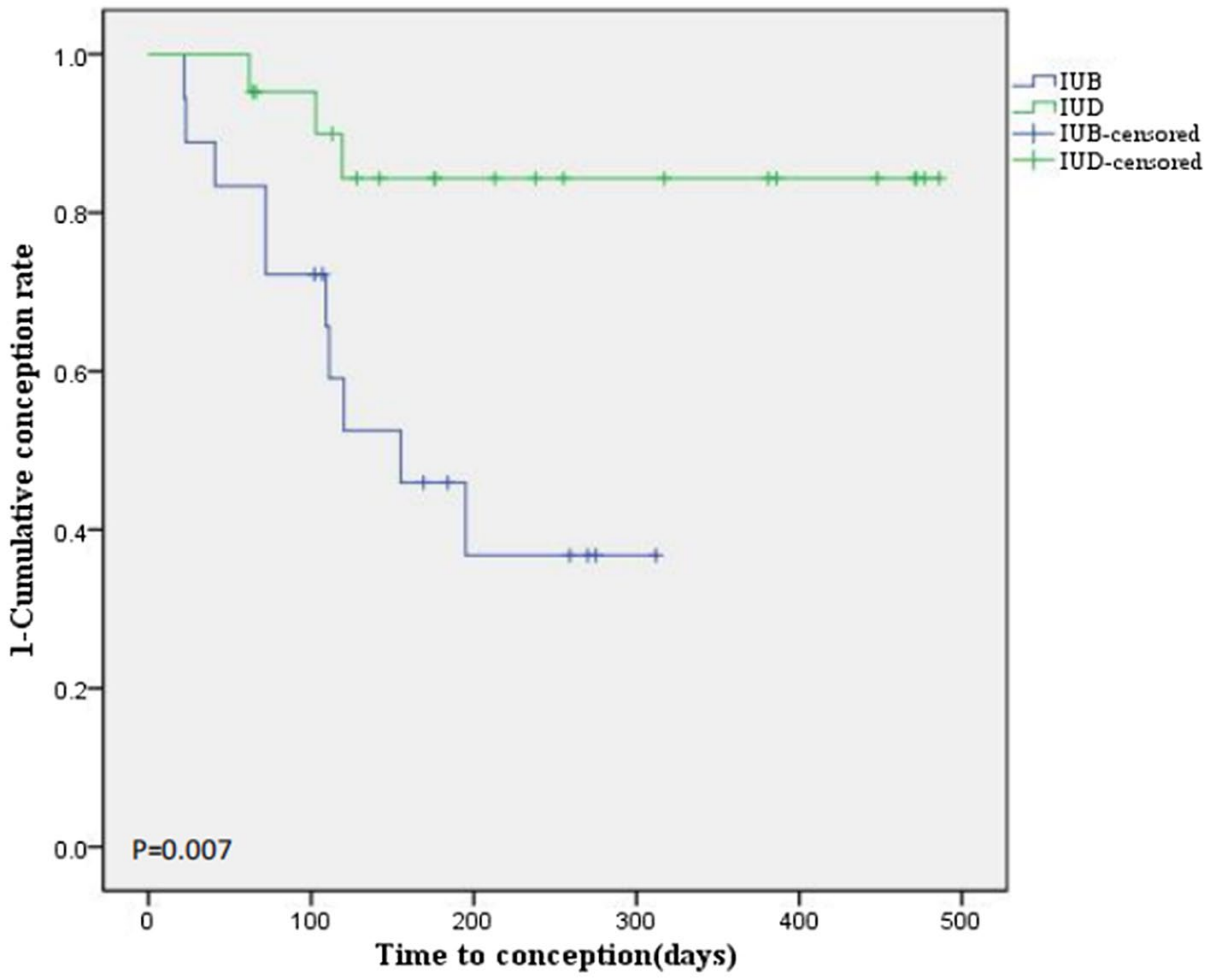
Cumulative conception rate after TCRA in IUA patients treated with IUB or IUD

Statistical data from this study showed that the intrauterine pregnancy rates of IUA patients in the IUB group and IUD group after treatment were 55.56% (10/18) and 14.29% (3/21), respectively, with a significant difference (Table 2, *P* = 0.015). The average time to conception of the two groups was 28.67 ± 10.69 days and 94.67 ± 29.40 days. As shown in Fig. 2, the cumulative conception rate of IUA patients after treatment in the IUB group was significantly higher than that in the IUD group (HR 0.201, 95% CI 0.055–0.734, *P* = 0.007).

## Discussion

### Principal findings

According to the results above, overall, IUA patients treated with the special IUB after TCRA had a better outcome than those treated with the IUD in that their readhesion rate was much lower and the adhesion severity was reduced much more significantly. However, both methods notably improved the menstrual volume, which might have also benefited from the utilization of sequential hormone therapy with estrogen and progesterone. Most importantly, patients with Asherman syndrome implanted with a special intrauterine balloon after surgery had a much higher intrauterine pregnancy rate than patients receiving an intrauterine contraception device after the TCRA procedure, particularly within two months after the last surgery. There was a significantly higher recurrence rate in the IUD group, so the time to conception after the last TCRA surgery was significantly longer.

### Results in the context of what is known

To a certain extent, an IUD can partially separate from the intrauterine wall and reduce readhesion. Randomized controlled trials (RCTs) found that for patients who received an IUD after TCRA, the overall conception rate and live birth rate were 27.5–47.2% and 20.0–28.0%, respectively [5, 12, 13], and the reformation rate was 35.0–43.1% [13, 14]. It was reported that 62.7% of IUA patients returned to normal menstruation in this way [2]. Given that intrauterine contraception devices have limited barrier surfaces, there is a higher readhesion rate. As revealed in this study analogously, the rate of menstrual volume increase was high. However, there were also much higher recurrence risks.

After TCRA, placement of an IUB can not only block the adhesion of different uterine walls but is also convenient for drainage of hematometra and inflammatory exudation in the uterine cavity, which contributes to minimizing the risk of infection and reducing the recurrence rate of intrauterine adhesions. Another study found that up to 81.4% of IUA patients had improved menstruation and were implanted with an IUB after surgery [2], the overall conception rate was 33.9–48.1%, and the recurrence rate of IUA after surgery was 13.6–38.7% [13–17]. Compared with placing an IUD after TCRA, insertion of an IUB can lead to a lower readhesion AFS score and a lower recurrence rate [2], which is entirely consistent with the results of this study.

In addition, preventing the recurrence of IUAs after surgery largely benefits from the use of estrogen in maintenance therapy, which can promote endometrial growth and regeneration to repair wounds. A randomized controlled study found that estrogen inhibited the reformation of adhesion and that the routine daily estrogen dose (2 mg) was sufficiently effective; there was no significant difference between the routine dose and a higher dose [18]. According to the results shown in this study, most IUA patients have a thin endometrium before TCRA; therefore, estrogen therapy is extremely important postoperatively and before conception.

There are two kinds of operating instruments for hysteroscopic adhesiolysis, namely, electric and nonelectric; the latter includes blunt dissection and cold scissors. As this study showed, for severe IUA patients, the nonelectric method was more effective than the electric method for preventing readhesion after TCRA. A meta-analysis also found that cold scissors are more efficient in preventing the recurrence of intrauterine adhesions [19].

Studies revealed that the incidence of CE was 35.4–46.28% in moderate and severe IUA patients, who had a higher recurrence rate and poorer reproductive outcome [8, 9]. In this hospital, operators sometimes diagnosed CE by visual signs during hysteroscopy, and consequently, IHC was optional in the pathological examination; hence, only a few IUA patients were diagnosed by postoperative pathology. An adequate course of antibiotic therapy is vitally important.

### Clinical implications

It is well known that trauma and infection are the main factors in the pathogenesis of IUAs, leading to endometrial ischemic changes and hypoxia, which promote associated endometrial repair deficiency and fibrosis signaling pathways or molecular mechanisms [20, 21]. A study found that the level of vascular endothelial growth factor (VEGF) was elevated due to vascular endothelial injury resulting from insufficient blood supply [22]. Meanwhile, as research has shown, estrogen receptors are increased because of estrogen deficiency in the local ischemic endometrium [23]. As an advantage of this study, a soft elastic saccule that significantly improves blood supply may be the reason for such a good therapeutic effect, which may upregulate many repair factors and downregulate certain proinflammatory factors and fibrosis promotion factors.

### Research implications

Although the clinical effect of placing an IUB after TCRA has been confirmed, it is still not completely suitable for the uterine cavity because of the shape, elasticity and compression of the balloon. Therefore, an IUB sometimes cannot adequately separate the wound surface, especially the wound surface of bilateral cornua uteri, and may affect the blood supply to the endometrium, which usually results in deficient repair and eventual necrosis. In this study, the special kind of IUB with many more soft elastic saccules was effective in the treatment of IUA patients after surgery. However, it is necessary to further search for the best volume of balloon and duration of IUB therapy in the uterine cavity. In addition, for IUA patients, we will further compare the sensitivity and specificity in the diagnostic criteria of CE by visual signs during hysteroscopy or postoperative pathologic results with immunohistochemistry staining.

### Strengths and limitations

As the results showed, for IUA patients, we demonstrated that using this kind of special IUB after TCRA had many more advantages. Meanwhile, we further revealed the importance of treatment with a cold knife, estrogen-progesterone therapy and antibiotics. However, we need to point out that due to the limitations of this study, the sample size was small and lacked adequate blinding. The treatment prognosis depends very much on the severity and extent of the adhesions, analyzing the definite classification of the location of IUAs could be much more significant [24]. However, due to a retrospective study, it is difficult to further clearly define classification. Furthermore, due to a short follow-up period, pregnancy outcomes were not revealed.

## Conclusions

In conclusion, the key to the treatment of IUA patients lies in the prevention of recurrence after TCRA. The results of this research suggested that patients in this special IUB group had better outcomes than patients in the IUD group. To prevent readhesion and improve the conception rate, IUB placement, the use of a cold knife, estrogen therapy and an adequate course of antibiotic therapy all have a positive influence. Owing to the small sample size in this study, the conclusions still require further validation. It is necessary to carry out a rigorous prospective study in a larger patient population or RCT to further define the efficacy and outcome of the special intrauterine balloon.

## Data Availability

The datasets used and/or analyzed during the current study are available from the corresponding author on reasonable request.

## Declarations

### Patient and public involvement

Patients were not involved in any aspect of the study design or conduct or in the development of the research question or outcome measures.
